# Shanghai Autism Early Development: An Integrative Chinese ASD Cohort

**DOI:** 10.1007/s12264-022-00904-y

**Published:** 2022-06-23

**Authors:** Yuan Dai, Yuqi Liu, Lingli Zhang, Tai Ren, Hui Wang, Juehua Yu, Xin Liu, Zilin Chen, Lin Deng, Minyi Tao, Hangyu Tan, Chu-Chung Huang, Jiaying Zhang, Qiang Luo, Jianfeng Feng, Miao Cao, Fei Li

**Affiliations:** 1 Department of Developmental and Behavioral Pediatric and Child Primary Care, Brain and Behavioral Research Unit of Shanghai Institute for Pediatric Research and Ministry of Education-Shanghai Key Laboratory for Children’s Environmental Health, Xinhua Hospital, Shanghai Jiao Tong University School of Medicine, Shanghai, 200092 China; 2 Center for Experimental Studies and Research, The First Affiliated Hospital of Kunming Medical University, Kunming, 650032 China; 3 Key Laboratory of Brain Functional Genomics (Ministry of Education), Affiliated Mental Health Center, School of Psychology and Cognitive Science, East China Normal University, Shanghai, 200062 China; 4 Shanghai Changning Mental Health Center, Shanghai, 200335 China; 5 State Key Laboratory of Cognitive Neuroscience and Learning, Beijing Normal University, Beijing, 100875 China; 6 Beijing Key Laboratory of Brain Imaging and Connectomics, Beijing Normal University, Beijing, 100875 China; 7 IDG/McGovern Institute for Brain Research, Beijing Normal University, Beijing, 100875 China; 8 National Clinical Research Center for Aging and Medicine at Huashan Hospital, Fudan University, Shanghai, 200433 China; 9 Institute of Science and Technology for Brain-Inspired Intelligence, Fudan University, Shanghai, 200433 China; 10 State Key Laboratory of Medical Neurobiology and Ministry of Education Frontiers Center for Brain Science, Institutes of Brain Science and Human Phenome Institute, Fudan University, Shanghai, 200032 China; 11 Key Laboratory of Computational Neuroscience and Brain-Inspired Intelligence, Ministry of Education, Fudan University, Shanghai, 200433 China

## Why Was the Cohort Set Up?

Autism spectrum disorder (ASD) is a child neurodevelopmental disorder, the onset of which is generally within 3 years of age, and often leads to lifelong impaired social and cognitive functions, which impose significant mental pressure and economic burdens on the family and society. In 2012, the incidence data released by the World Health Organization showed that the global prevalence of ASD was ~0.625% [1], and the latest report from the Centers for Disease Control and Prevention show that the incidence of ASD in the USA had reached 2.3% in 2021 [2]. According to a nationwide survey of over 120,000 children aged 6–12 years, the prevalence of ASD in China was reported to be 0.7% in 2020 [3]. The mission to improve the screening, diagnosis, rehabilitation, and assistance of ASD children aged 0–6 years has been listed in the national strategic deployments for the next decade, including the Outline for the Development of Children in China (2021–2030) and the China Brain Project 2030.

Although ASD is highly heritable, the biological underpinnings and development over the lifespan remain largely unclear due to its heterogeneity. To address these challenges, large-scale sample collections focusing on the early developmental period of ASD are essential, as these are critical for identifying ways of screening high-risk populations, determining the diagnosis at earlier ages, selecting optimal treatments, and predicting outcomes.

**Fig. 1:**
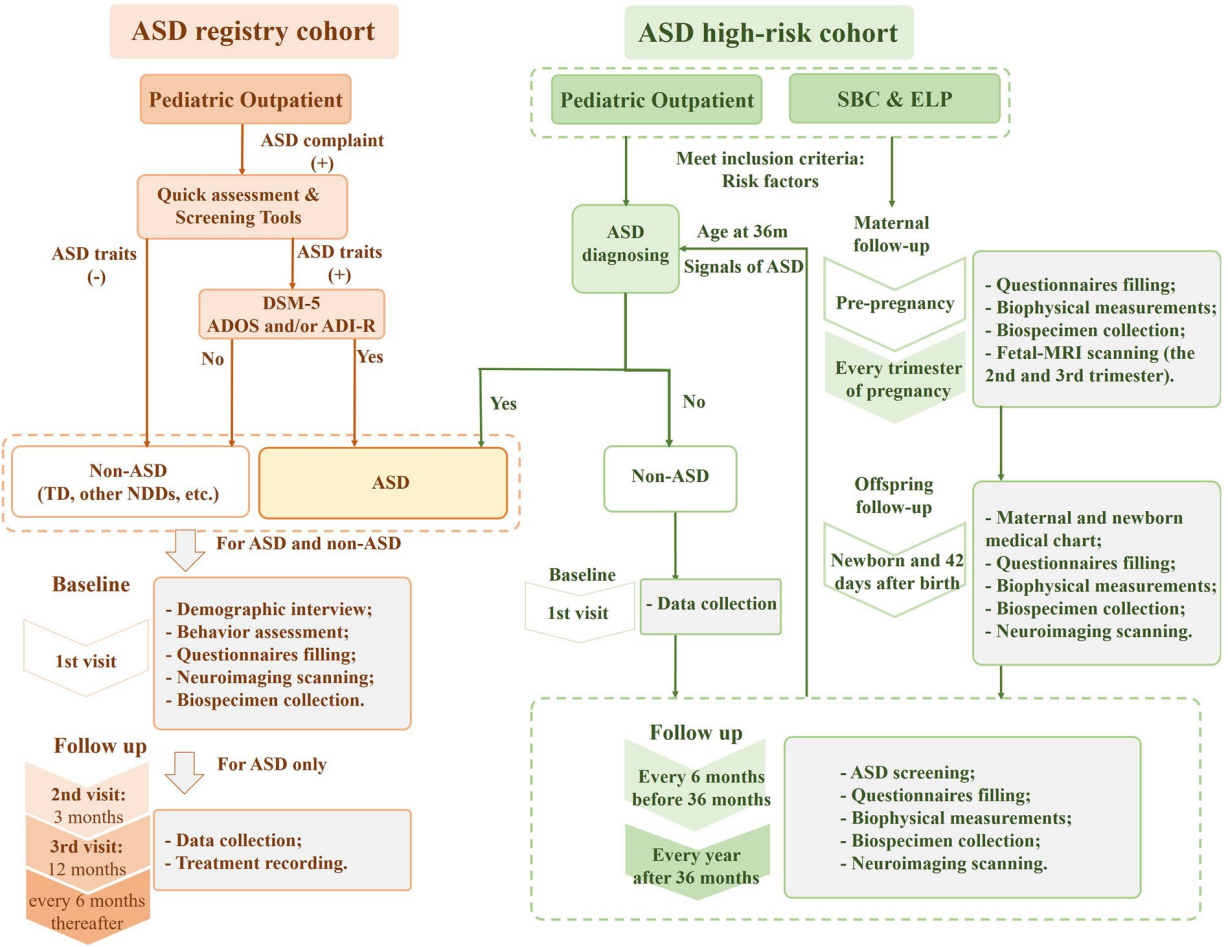
Study design and multi-dimensional measurements.

The Shanghai Autism Early Developmental (SAED) Cohort was established with the support of several foundations, including major projects of the Shanghai Science and Technology Commission, the Natural Science Foundation of China, and the Science and Technology Commission of Shanghai Municipality. Some of the data also represent important parts of the Zhangjiang International Brain Biobank [4]. Unique biological samples serially collected throughout pregnancy and childhood with early screening, diagnostic assessments, and re-assessments to identify antenatal, perinatal, and early postnatal risk and protective factors. Brain connectomic, genetic, proteomic, immunological, metagenomic, and microbiological tools will be applied to provide a rich, longitudinal view of ASD trajectories. Figure 1 illustrates the design of the study. This cohort aims to facilitate ASD research both in China and world-wide.

## Who Is in the Cohort?

### The ASD Registry Cohort

The parents of children who visited the Xinhua Hospital affiliated to Shanghai Jiao Tong University School of Medicine with concerns about language delay and/or social deficits were interviewed by developmental-behavioral pediatricians who applied two quick assessments with the following criteria. The “five-no/less” early signs of autism including [5] (1) problems with eye contact, (2) no/less response to their name, (3) problems following another person’s gaze or pointed finger to an object (or “joint attention”), (4) poor skills in pretend play and imitation, and (5) problems with nonverbal communication; as well as an abnormal developmental trajectory of social skills and communication. Meanwhile, early screening tools for ASD were applied to different age groups: the warning signs checklist of child psychology and behavior developmental problems, the Modified Checklist for Autism in Toddlers—Revised, the Clancy Behavior Scale, the Autism Behavior Checklist, and the Social Responsiveness Scale. Children who were considered to be at high risk of ASD after initial assessment were further evaluated by assessors using diagnostic instruments: the Autism Diagnostic Observation Schedule and/or the Autism Diagnostic Interview—Revised. The developmental-behavioral pediatricians made a final diagnosis of ASD according to Diagnostic and Statistical Mental Disorders, fifth edition (DSM-5). Non-ASD groups included neurotypical children and children with other neurodevelopmental disorders (e.g., developmental delay/intellectual disability, or attention deficit hyperactivity disorder (ADHD)) according to DSM-5, none of whom met the criteria for ASD. Children in this cohort were mainly 3–12 years old.

### The ASD High-Risk Cohort

Children in this cohort were recruited from pediatric outpatients and risk populations from the Shanghai Birth Cohort included in the Early Life Plan (SBC & ELP) [6] who displayed at least one of following cues: (1) inherited risk, which refers to having an elder/younger sibling with ASD and/or a family history of schizophrenia, mood disorders, or any other psychiatric disorders and behavioral problems [7]; (2) maternal adverse events associated with autism (e.g., maternal metabolic disease [8–10], autoimmune disease [11], or cardiovascular disease [12]); (3) structural abnormalities found during fetal brain MRI scanning (e.g., lateral ventricular widening [13, 14]); (4) adverse birth events (e.g., preterm birth [15]); (5) early medical history after birth related to the nervous system (e.g. neonatal hypoxia [16]); and (6) adverse environmental exposure after birth (e.g., excessive exposure to heavy metals such as lead and mercury [17], and early-life digital media exposure [18]). Notably, children exposed to other newly-identified risk factors will also be recruited in the future.

## How Often Have They Been Followed Up?

In the ASD registry cohort, participants who met the inclusion criteria and were willing to participate in the project were recruited during their first visit to Xinhua Hospital for conventional diagnosis. They were welcomed to come to the study office for the second visit after 3–6 months, the third visit after 1 year, and visits every 6 months thereafter. Children who did not meet the diagnostic criteria of ASD after treatment during follow-up were classified as children once diagnosed with ASD.

In the ASD high-risk cohort, outpatient participants who met the inclusion criteria and were willing to participate were recruited during their first visit to Xinhua Hospital. Participants from the SBC & ELP were recruited and followed up as described in [6]. Specifically, all participants were first interviewed and screened for ASD traits at 6 months old and asked to visit for follow-up at every 6 months. Those who were diagnosed with ASD during early detection were transferred to the ASD registry cohort.

## What Has Been Measured?

From all participants in the ASD registry cohort at their first visit, we collected information using demographic interviews (e.g., nationality, sex, age, perinatal conditions, and socio-economic conditions), behavior evaluation, questionnaires completed by guardians, neuropsychological tests, neuroimaging scans [e.g. magnetic resonance imaging (MRI), electroencephalogram (EEG), and functional near-infrared spectroscopy (fNIRS)], and biological samples, including blood, stool, and urine. Specifically, the scales shown in Table S1 were applied to evaluate the core symptoms of ASD, cognition, and the symptoms of comorbidities (e.g., ADHD, sleep disorders, or mood disorders). To measure the conditions of the participants’ parents, we also asked the parents to complete the questionnaires listed in Table S1. Questionnaires about protective factors, such as folic acid intake and parenting style [19] were also included. The brain imaging and biospecimen data were collected and processed by specially-trained staff and stored at the Xinhua Bio-bank. For the participants’ follow-up visits, data similar to that in the baseline visit and information regarding behavioral interventions or drug treatments were recorded.

In the ASD high-risk cohort, the same measurements as for the ASD registry cohort were collected from participants recruited from the SBC & ELP when their offspring reached the age of 36 months and from outpatient participants when they first visited Xinhua Hospital. At each visit during pregnancy, participants from the SBC & ELP were subjected to questionnaires, biophysical measurements, and biospecimen collections, including demographic characteristics, environmental exposure, housing characteristics, chemical exposure, use of pesticides, occupational exposure, social support, health behavior, diet, medical history, health status, venous blood, urine, and environmental pollutants (i.e., perfluoroalkyl and polyfluoroalkyl substances, phenols, organophosphate and pyrethroid pesticides, heavy metals, micronutrients, and antibiotics). Fetal head MRI imaging data were collected during the second and third trimesters, when brain ventricular enlargement was screened by ultrasound. After delivery, we conducted maternal and newborn medical chart abstraction, biophysical measurements, and physical examination of the child. Biospecimen and questionnaire data were collected including the placenta, cord blood, mother’s hair and nails, child’s urine and blood, feeding, diet, sleep, and environmental exposure.

## How Far Are We Going?

Up to March, 2022, the ASD registry cohort, which started in 2015, has recruited 1,091 ASD participants (age range: 2.00–14.50 years) and 113 non-ASD participants (age range: 1.26–11.88 years). Data collection from the ASD high-risk cohort started from 2013 and has recruited 1,278 participants (212 for inherited risk, 205 for lateral ventricular widening, 661 for gestational diabetes mellitus, and 200 for preterm birth).

Specifically, MRI imaging data were collected for most participants in the cohort, including T1-weighted and T2-weighted structural, resting-state functional, and diffusion weighted MRI data. The structural images of each participant were inspected by two experienced radiologists from Xinhua Hospital, and no abnormalities were found in any participant’s structural images. EEG and fNIRS data were also collected from children who could not tolerate MRI or for specific research purposes.

## What Are the Main Strengths and Weaknesses?

Our cohort has several strengths. First, it is a single-center study with a large sample size, which makes the multi-modality data comparable. Second, multi-modality data enable multiomics analysis, thus supporting research into the neurobiological basis of ASD. Besides, this cohort is open and non-fixed, which allows adjustment based on new findings. Protocols are designed to establish multi-center studies with a unified design in the future, to identify the specific factors for ASD etiological screening, and to formulate prevention and control strategies in various regions. Finally, the pregnant cohort allows us to determine the risk and protective factors, mechanisms, and markers associated with ASD. Comparisons between the SAED cohort and other ASD cohorts are presented in Table S2.

Some limitations should also be stated. This cohort was limited mainly to residents of Shanghai, Zhejiang, and Jiangsu in order to maintain long-term follow-up. Although the cohort covers both suburban and urban populations, few participants living in rural settings have been enrolled due to the study location. Furthermore, the study population was recruited from outpatient clinics, which means the ASD-related population unwilling to seek medical care has not been included. These issues limit the cohort’s ability to represent the entire ASD-related population.

## Supplementary Information

Below is the link to the electronic supplementary material.

Supplementary file1 (PDF 203 KB)
