# Supplementary material for: Shanghai Autism Early Development: An Integrative Chinese ASD Cohort
Source: Neurosci Bull. 2022 Jun 23;38(12):1603–7. doi: 10.1007/s12264-022-00904-y (PMC9723093; doi:10.1007/s12264-022-00904-y)
Supplement: Supplementary file 1 — Supplementary file1 (PDF 203 KB) [file 12264_2022_904_MOESM1_ESM.pdf]

Supplementary Information

Table S1. Summary of measurements in the SAED study.

		Domain/task	Time point	Schedule			
ASD-screening				A	B	C	D
	OA	Modified Checklist for Autism in Toddlers–Revised	Base	P	-	-	-
	OA	Clancy Behavior Scale	Base	P	P	P	-
	OA	Autism Behavior Checklist	Base & FU	P	P	P	P
	OA	Social Responsiveness Scale	Base & FU	-	P	P	P
Demographic							
	OA	Demographic characteristics	Base & FU	P	P	P	P
Physical growth							
	IA	Height, weight, head circumference	Base & FU	S	S	S	S
Clinical diagnosis							
	IA	Autism Diagnostic Interview–Revised	Base	P	P	P	P
	IA	Autism Diagnostic Observation Schedule	Base & FU	S	S	S	S
Dimensional measures of ASD symptoms							
	OA	Social Responsiveness Scale	Base & FU	-	P	P	P
	OA	Repetitive Behavior Scale–Revised	Base & FU	P	P	P	P
	OA	Short Sensory Profile	Base & FU	P	P	P	P
	OA	Autism Behavior Checklist	Base & FU	P	P	P	P
	OA	Sensory Experiences Questionnaire–short version 3.0	Base & FU	P	P	P	P
	IA	Clinical Global Impression	FU	P	P	P	P
Comorbidities							
	OA	DSM-5 ADHD rating scale	Base & FU	-	-	P	P
	OA	Strengths and Difficulties Questionnaire	Base & FU	-	P	P	P
	OA	Behavior Rating Inventory of Executive Function	Base & FU	P	P	P	P
	OA	Screen for Child Anxiety Related Emotional Disorders	Base & FU	-	-	-	P
	OA	Depression Self-rating Scale for Children	Base & FU	-	-	-	P
	OA	Child Behavior Checklist	Base & FU	P	P	P	P
Quality of life behavior							
	OA	Social Adaptation Scale for Infants–Junior Middle School Students Revised by Qihua Zuo	Base & FU	P	P	P	P
Medical or psychiatric history							
	IA	Individual Medical History Interview	Base & FU	P	P	P	P
	IA	Family Medical History Interview	Base & FU	P	P	P	P
Cognitive and psychological profile							
	IA	Gesell Developmental Schedules	Base & FU	S	S	-	-
	IA	Wechsler Preschool & Primary Scale of Intelligence	Base & FU	-	-	S	-
	IA	Wechsler Intelligence Scale for Children–Revised	Base & FU	-	-	-	S
	IA	Symbolic Play Test	Base & FU	S	S	S	-
	IA	A Developmental Neuropsychological Assessment–Second Edition: Social Perception battery	Base & FU	-	-	-	Sa
	IA	Continuous Performance task	Base & FU	-	-	-	Sa
Environmental exposure							
	IA	Physical and chemical exposure questionnaire	Base & FU	P	P	P	P
	OA	Parents' Parenting Behavior Questionnaire	Base	P	P	P	P
	OA	Protective factors questionnaire	Base & FU	P	P	P	P
Treatment information							
	IA	Information form for intervention	Base & FU	P	P	P	P
	IA	Information form for drug-treatment	Base & FU	P	P	P	P
Neuroimaging							
	IA	Structural MRI	Base & FU	S	S	S	S

	IA	FLAIR sequence or localizer sequence MRI	Base & FU	S	S	S	S
	IA	Diffusion-weighted imaging	Base & FU	S	S	S	S
	IA	Resting-state functional MRI	Base & FU	S	S	S	S
EEG							
	IA	Resting state	Base & FU	S	S	S	S
	IA	Dyadic parent-child interaction	Base & FU	S & P	S & P	S & P	S & P
fNIRS							
	IA	Resting state	Base & FU	-	S	-	S
	IA	Go/No-Go task	Base & FU	-	S	-	S
Biological samples							
	IA	Blood sample (for multi-omics analyses)	Base & FU	S	S	S	S
	IA	Urine (for biochemical biomarkers)	Base & FU	S	S	S	S
	IA	Stool (for intestinal microflora study)	Base & FU	S	S	S	S
Assessment of clinical symptoms in both biological parents							
	OA	Autism Quotient, adult version	Base & FU	P	P	P	P
	OA	Adult ADHD Self-Report Scale	Base & FU	P	P	P	P
	OA	Self-Rating Anxiety Scale	Base & FU	P	P	P	P
	OA	Center for Epidemiological Studies–Depression	Base & FU	P	P	P	P

*ADHD: attention-deficit hyperactivity disorder, ASD: autism spectrum disorder, Base: baseline assessment wave, DSM: Diagnostic and Statistical Manual of Mental Disorders, FU: follow-up assessment wave, IA: investigator administered assessment at the institute and/or online, MRI: magnetic resonance imaging, OA: online assessment, P reported by parent, S: self-participating

* Schedule A: children aged 0–3 years;
Schedule B: children aged 3–4 years;
Schedule C: children aged 4–6 years;
Schedule D: children and adolescents aged 7–12 years. a: not applicable for children with IQ <70.

Table S2. Comparisons between the SAED Cohort and current ASD cohorts.

	**Shanghai Autism Early Developmental Cohort (SAED Cohort)**	**Autism Brain Imaging Data Exchange (ABIDE I & II)**	**Infant Brain Imaging Study**	**EU-AIMS Longitudinal European Autism Project**	**Baby Siblings Research Consortium**	**SFARI Simons Simplex Collection**
**Age-range**	ASD<br>2.00–14.50 years<br>Non-ASD<br>1.26–11.88 years	ABIDE I<br>7–64 years;<br>ABIDE II<br>5–64 years	First visit at 6 months	ASD<br>6.08–30.60 years<br>TD/ID<br>6.24–30.78 years	First visit at 6 months	4–18 years
**Data**	Head-MRI;<br>EEG;<br>fNIRS;<br>Behavioral assessments and questionnaires;<br>Biological samples	Head-MRI;<br>Behavioral assessments.	Head-MRI;<br>EEG;<br>Behavioral assessments and questionnaires;<br>LENA language recording	Head-MRI;<br>EEG;<br>Behavioral assessments and questionnaires;<br>Biological samples	Behavioral assessments	Blood sample
**Sample size**	**ASD registry cohort**<br>ASD ⩾1500<br>Non-ASD ⩾ 500<br>**ASD high-risk cohort**<br>Inherited risks (n ⩾ 500): children with ASD siblings, etc.;<br>Environmental risks (n ≥500): gestational diabetes mellitus; preterm birth, excessive heavy metals exposure, etc.;<br>Fetal brain abnormality (n ⩾ 500): lateral ventricular widening, etc.	**ABIDE I**<br>ASD (n = 539);<br>Controls (n = 573);<br>**ABIDEII**<br>ASD (n = 521)<br>Controls (n = 593).	**ASD High-risk** (n=186)<br>Infants under 6 months with ASD sibling;<br>**ASD Low-risk** (n = 76)<br>Infants with typical development sibling	ASD (n = 437);<br>TD/ID (n = 300);<br>A further twin cohort (n = 102, including monozygotic or dizygotic twin pairs discordant for ASD (n = 36)	**ASD High-risk** (n = 1241)<br>Infants under 6 months with ASD sibling;<br>**ASD Low-risk** (n = 583)<br>Infants with typical development sibling	ASD trios (n = 358);<br>male siblings (n = 709);<br>820 siblings (n = 820).<br>Simplex families (n = 2,600).
**Centers**	Single center	Multicenter	Multicenter	Multicenter	Multicenter	Multicenter
**Treatment**	Yes	No	No	No	No	No
**Follow-up**	**ASD registry cohort**<br>Second visit after 3–6 months;<br>Third visit after 1 year;<br>then visits every 6 months thereafter<br>**ASD high-risk cohort**<br>Visits at 6, 12, 18, 24, 30, and 36 months and every year thereafter	No	Visits at 6, 9, 12, 18, and 24 months	Second visit after 12–24 months	Visits at 6, 9, 12, 15, 18, 24, and 36 months	No

Clinical scales	ASD core symptoms; Comorbidities; Quality of life behavior; Cognitive and psychological profile; Medical or psychiatric history; Assessment of clinical symptoms and cognition in both biological parents; Environmental exposure; Treatment information; ASD screening	ASD core symptoms; Cognitive and psychological profile	ASD core symptoms; Quality of life/adaptive behavior; Cognitive and psychological profile; Medical or psychiatric history	ASD core symptoms; Comorbidities; Quality of life/adaptive behavior; Cognitive and psychological profile; Medical or psychiatric history; Assessment of clinical symptoms and cognition in both biological parents	ASD core symptoms; Quality of life/adaptive behavior; Cognitive and psychological profile	ASD core symptoms; Quality of life/adaptive behavior; Cognitive and psychological profile
